# Supplementary material for: Colonic epithelial regeneration shapes susceptibility to Clostridioides difficile infection
Source: bioRxiv. 2026 May 27:2026.05.21.727036. Preprint. [Version 2] doi: 10.64898/2026.05.21.727036 (PMC13228475; doi:10.64898/2026.05.21.727036)
Supplement: Supplement 11 [file NIHPP2026.05.21.727036v2-supplement-11.pdf]

bioRxiv preprint doi: https://doi.org/10.64898/2026.05.21.727036; this version posted May 27, 2026. The copyright holder for this preprint (which was not certified by peer review) is the author/funder, who has granted bioRxiv a license to display the preprint in perpetuity. It is made available under a CC-BY 4.0 International license.

(

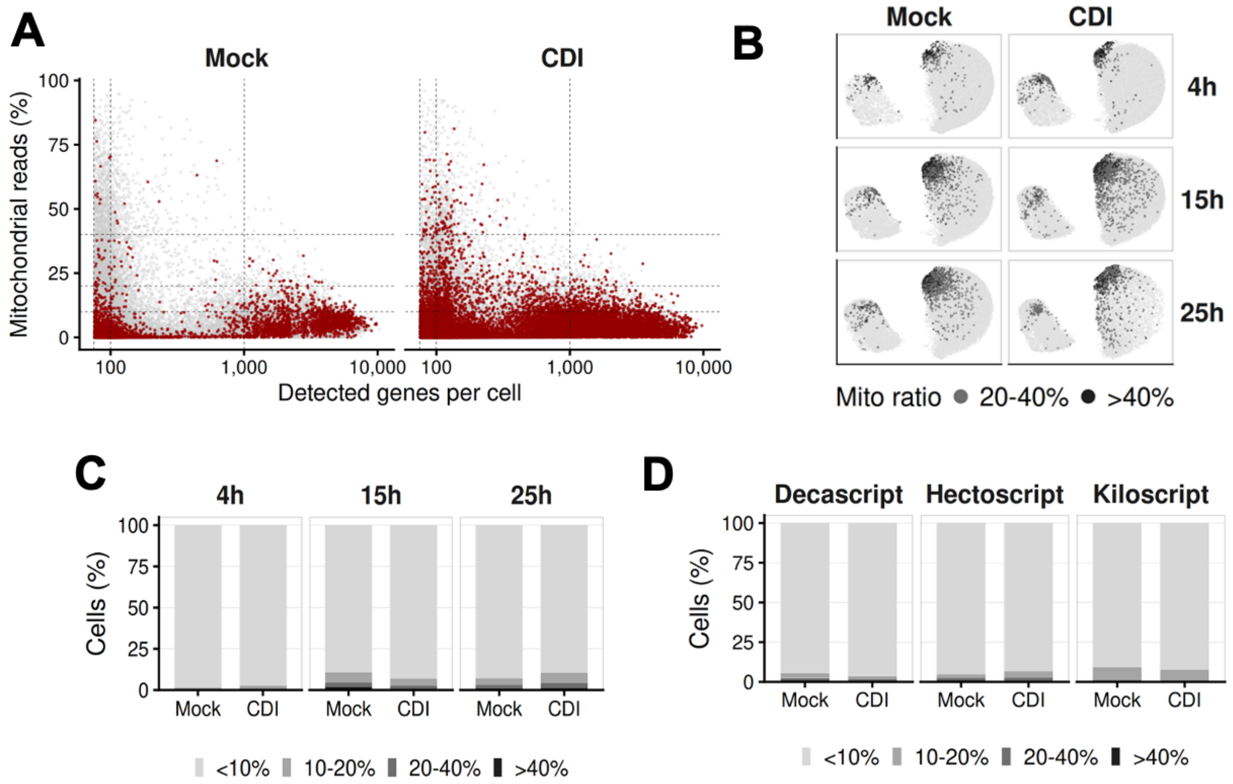

Figure S1. Characterization of single-cell profiles across mitochondrial read fraction and transcript complexity, related to Figure 1

(A) Distribution of detected genes per cell profile and mitochondrial read fraction in mock and CDI colonoid samples. Cells with high (top 10%) CDI-associated inflammatory (*CXCL8*, *TNF*, *IL1B*, and *IL15*) module scores are colored red. Horizontal dashed lines indicate commonly used mitochondrial read fraction filtering thresholds.

(B) UMAP visualization of integrated colonoid epithelial cells colored by mitochondrial read fractions (20–40% and >40%). UMAP projection stratified by CDI status and timepoint at 4, 15, 25 h post inoculation.

(C) Distribution of mitochondrial read fractions across timepoints and CDI status.

(D) Distribution of mitochondrial read fractions across transcript-count classes (Decascript, Hectoscript, and Kiloscript) in Mock and CDI samples.

bioRxiv preprint doi: https://doi.org/10.64898/2026.05.21.727036; this version posted May 27, 2026. The copyright holder for this preprint (which was not certified by peer review) is the author/funder, who has granted bioRxiv a license to display the preprint in perpetuity. It is made available under a CC-BY 4.0 International license.

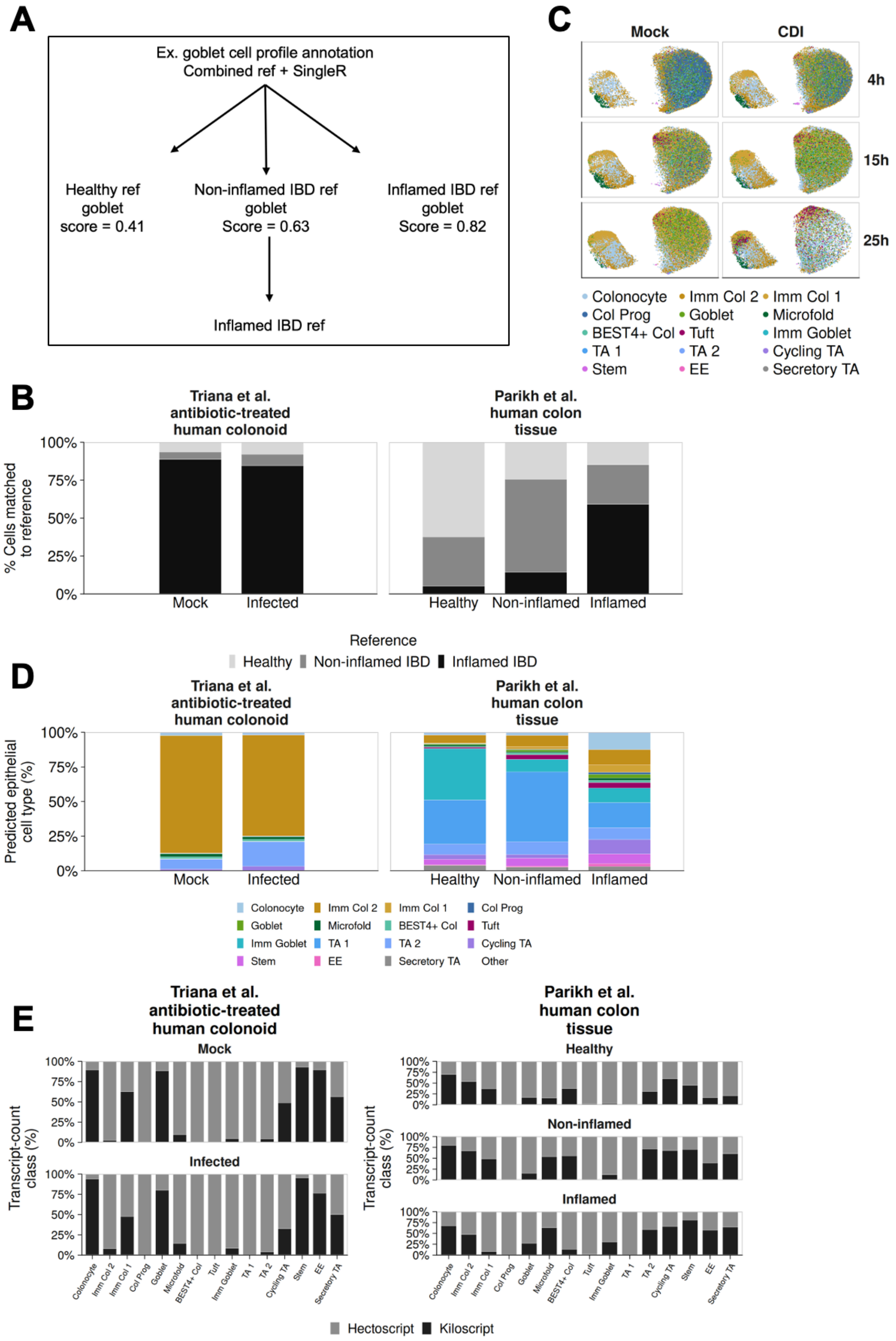

bioRxiv preprint doi: https://doi.org/10.64898/2026.05.21.727036; this version posted May 27, 2026. The copyright holder for this preprint (which was not certified by peer review) is the author/funder, who has granted bioRxiv a license to display the preprint in perpetuity. It is made available under a CC-BY 4.0 International license.

Figure S2. Disease-contextual epithelial type annotation and external dataset validation, related to Figure 2
(A) Schematic of the cell type annotation workflow used to identify reference type for final predictions. SingleR annotation was first performed using a combined atlas of epithelial single-cell profiles from healthy, non-inflamed IBD, and inflamed IBD human colon tissue from Smillie et al. Example similarity scores for a query goblet cell profile are shown schematically. The dominant disease-state reference is identified for each dataset and then used for downstream epithelial cell type annotation.
(B) Reanalyzed external datasets used to validate method for reference based cell type annotation. Triana et al. antibiotic-treated human colonoids and Parikh et al. human colon tissue samples were annotated using Singler with the combined reference. Bars represent proportions of cells annotated by each disease-state reference for each sample.
(C) UMAP visualization of all samples in this study integrated. Points representing single-cell profiles are colored by cell types annotated using the inflamed IBD colon reference. UMAP projection stratified by CDI status and timepoint at 4, 15, 25 h post inoculation.
(D) SingleR-predicted cell-type composition in external validation datasets following disease-state reference cell type annotation. Bars represent the proportion of cells assigned to each epithelial subtype within each sample.
(E) Distribution of transcript-count classes across predicted epithelial cell types in external validation datasets. Bars represent the proportion of Hectoscript and Kiloscript cells within annotated epithelial subtypes for each sample.

bioRxiv preprint doi: https://doi.org/10.64898/2026.05.21.727036; this version posted May 27, 2026. The copyright holder for this preprint (which was not certified by peer review) is the author/funder, who has granted bioRxiv a license to display the preprint in perpetuity. It is made available under a CC-BY 4.0 International license.

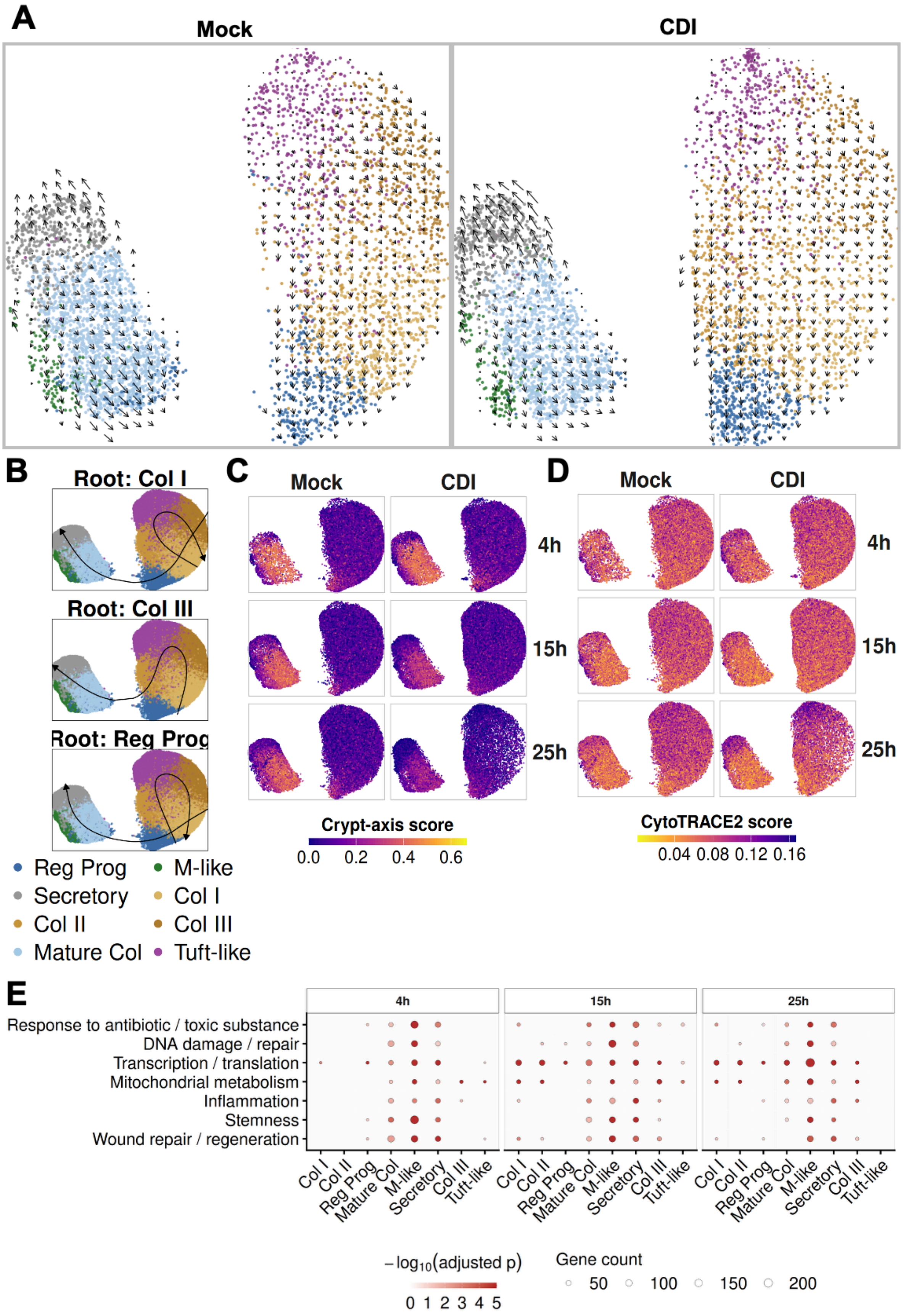

bioRxiv preprint doi: https://doi.org/10.64898/2026.05.21.727036; this version posted May 27, 2026. The copyright holder for this preprint (which was not certified by peer review) is the author/funder, who has granted bioRxiv a license to display the preprint in perpetuity. It is made available under a CC-BY 4.0 International license.

Figure S3. Crypt-axis gradients, CytoTRACE2 scores, lineage structure, and RNA velocity across Mock and CDI colonoids, related to Figure 3

(A) RNA velocity streamlines overlaid on UMAP embeddings for Mock and CDI colonoids at each timepoint (4 h, 15 h, 25 h). Arrows indicate the direction and magnitude of inferred transcriptional change. Cells are colored by cell state.

(B) Lineage inference in Mock samples using alternative root clusters (Col I, Col III, and regenerative progenitor). Inferred trajectories are projected onto the UMAP manifold with arrows indicating inferred directionality.

(C) UMAP visualization of integrated colonoid epithelial cells colored by crypt-axis score and shown separately by condition (Mock, CDI) and timepoint (4 h, 15 h, 25 h).

(D) UMAP visualization of integrated colonoid epithelial cells colored by CytoTRACE2 score and shown separately by condition (Mock, CDI) and timepoint (4 h, 15 h, 25 h).

(E) Representative Gene Ontology (GO) categories enriched among genes identified in mock-treated epithelial states across timepoints that were differentially expressed in the same direction in both replicates. Dot size indicates the number of overlapping genes and color intensity represents −log10 adjusted p value.

bioRxiv preprint doi: https://doi.org/10.64898/2026.05.21.727036; this version posted May 27, 2026. The copyright holder for this preprint (which was not certified by peer review) is the author/funder, who has granted bioRxiv a license to display the preprint in perpetuity. It is made available under a CC-BY 4.0 International license.

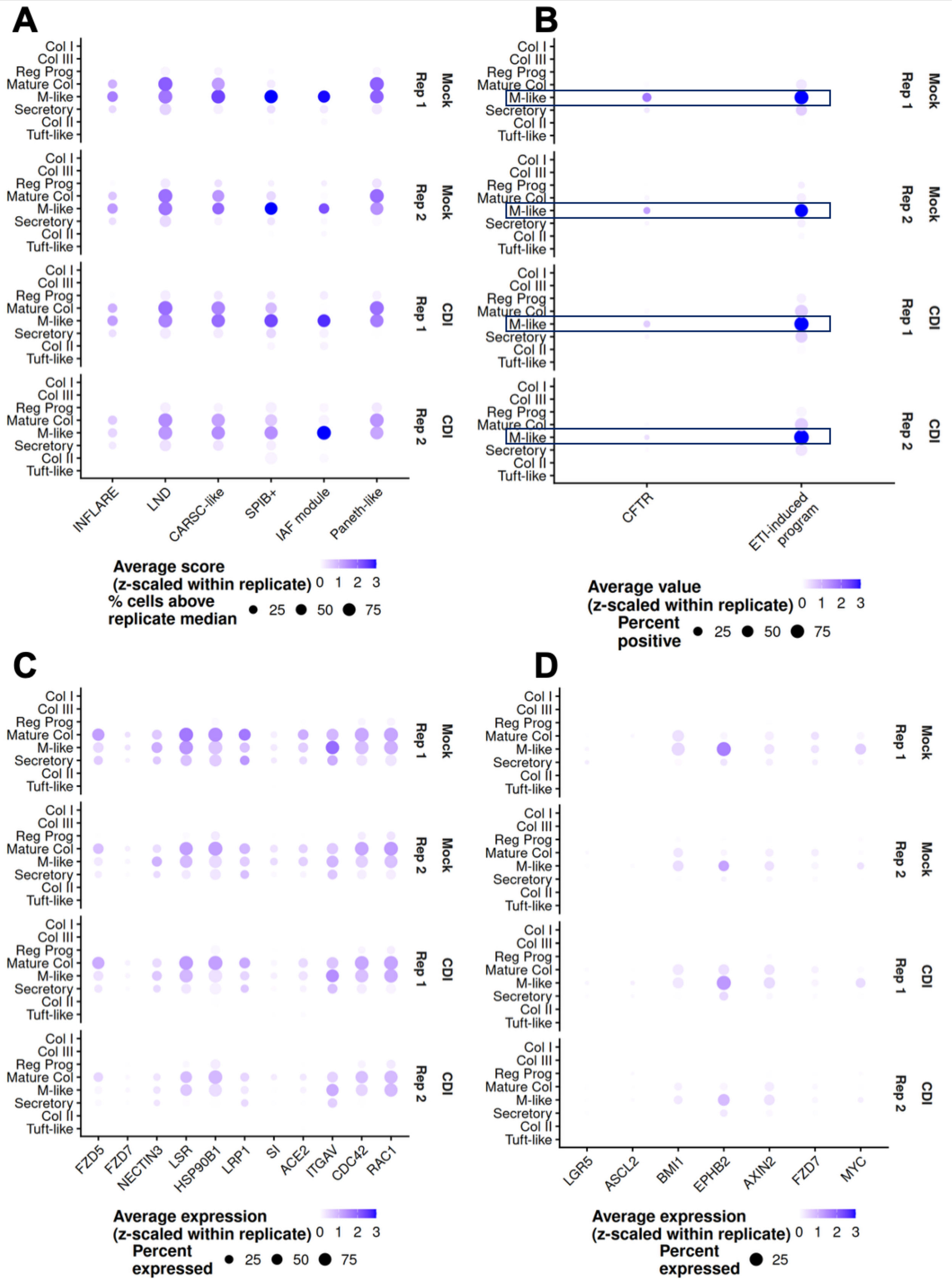

bioRxiv preprint doi: https://doi.org/10.64898/2026.05.21.727036; this version posted May 27, 2026. The copyright holder for this preprint (which was not certified by peer review) is the author/funder, who has granted bioRxiv a license to display the preprint in perpetuity. It is made available under a CC-BY 4.0 International license.

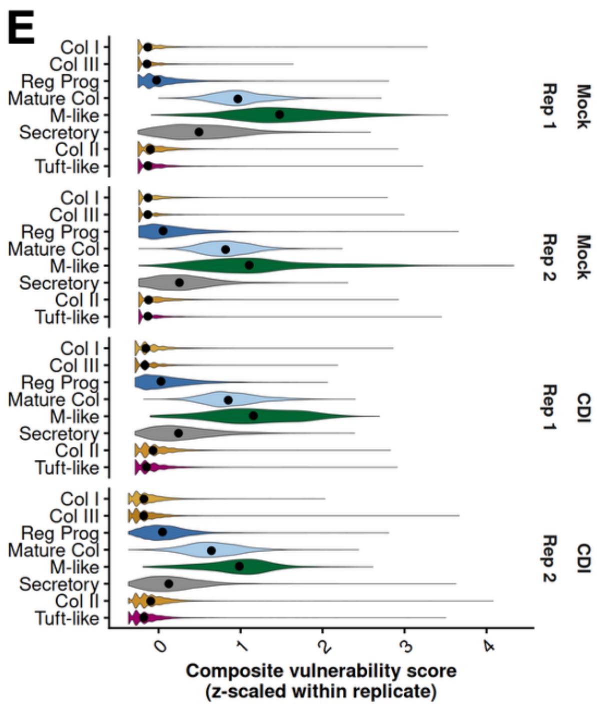

Figure S4. Regenerative, CFTR-associated, and host-interaction programs across epithelial cell states in Mock and CDI colonoid replicates, related to Figure 4

(A) Dot plot of regenerative and inflammation-associated transcriptional programs across epithelial cell states in Mock and CDI replicate samples. Dot size represents the percentage of cells above the replicate-specific feature median and color indicates average score (z-scaled within replicate).

(B) Dot plot of CFTR expression and ETI-induced transcriptional program scores across epithelial cell states in Mock and CDI replicate samples. Dot size represents the percentage of positive cells within each biological replicate and color indicates average expression (z-scaled within replicate).

(C) Dot plot of host-interaction genes across epithelial cell states in Mock and CDI conditions. Genes include C. difficile toxin receptors and host-interaction factors (FZD5, FZD7, NECTIN3, LSR, HSP90B1, LRP1, SI) together with epithelial interaction genes associated with other pathogens (ACE2, ITGAV, CDC42, RAC1). Dot size represents the percentage of positive cells within each biological replicate and color indicates average expression (z-scaled within replicate).

(D) Dot plot of stem-cell and Wnt signaling markers across epithelial cell states in Mock and CDI replicate samples. Markers were selected from Mileto et al. study of CDI-associated stem-cell damage and epithelial repair dysfunction. Dot size represents the percentage of positive cells within each biological replicate and color indicates average expression (z-scaled within replicate).

(E) Violin plots of composite vulnerability scores across epithelial cell states shown separately for Mock and CDI samples. Scores were normalized independently within biological replicates before visualization. Points represent replicate–timepoint medians (4 h, 15 h, 25 h).

bioRxiv preprint doi: https://doi.org/10.64898/2026.05.21.727036; this version posted May 27, 2026. The copyright holder for this preprint (which was not certified by peer review) is the author/funder, who has granted bioRxiv a license to display the preprint in perpetuity. It is made available under a CC-BY 4.0 International license.

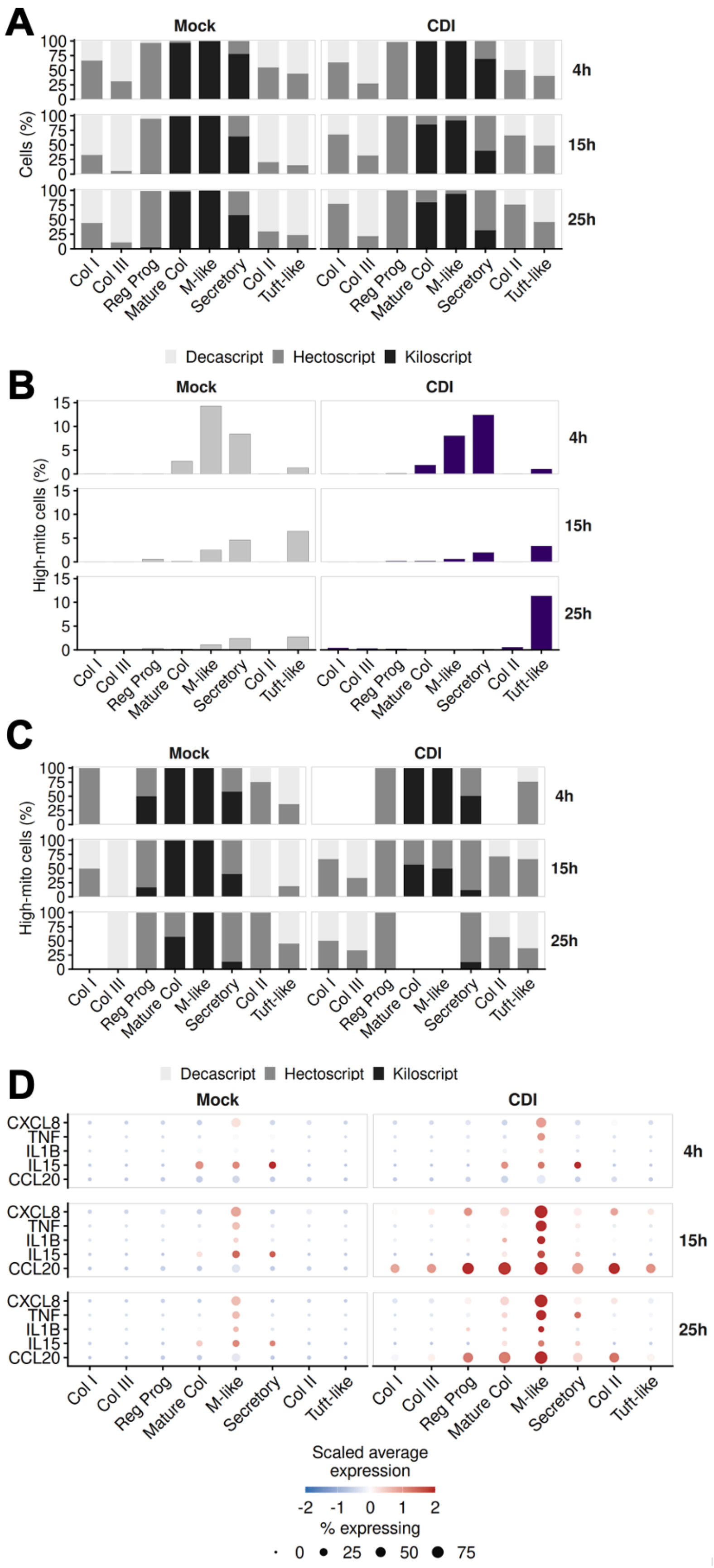

bioRxiv preprint doi: https://doi.org/10.64898/2026.05.21.727036; this version posted May 27, 2026. The copyright holder for this preprint (which was not certified by peer review) is the author/funder, who has granted bioRxiv a license to display the preprint in perpetuity. It is made available under a CC-BY 4.0 International license.

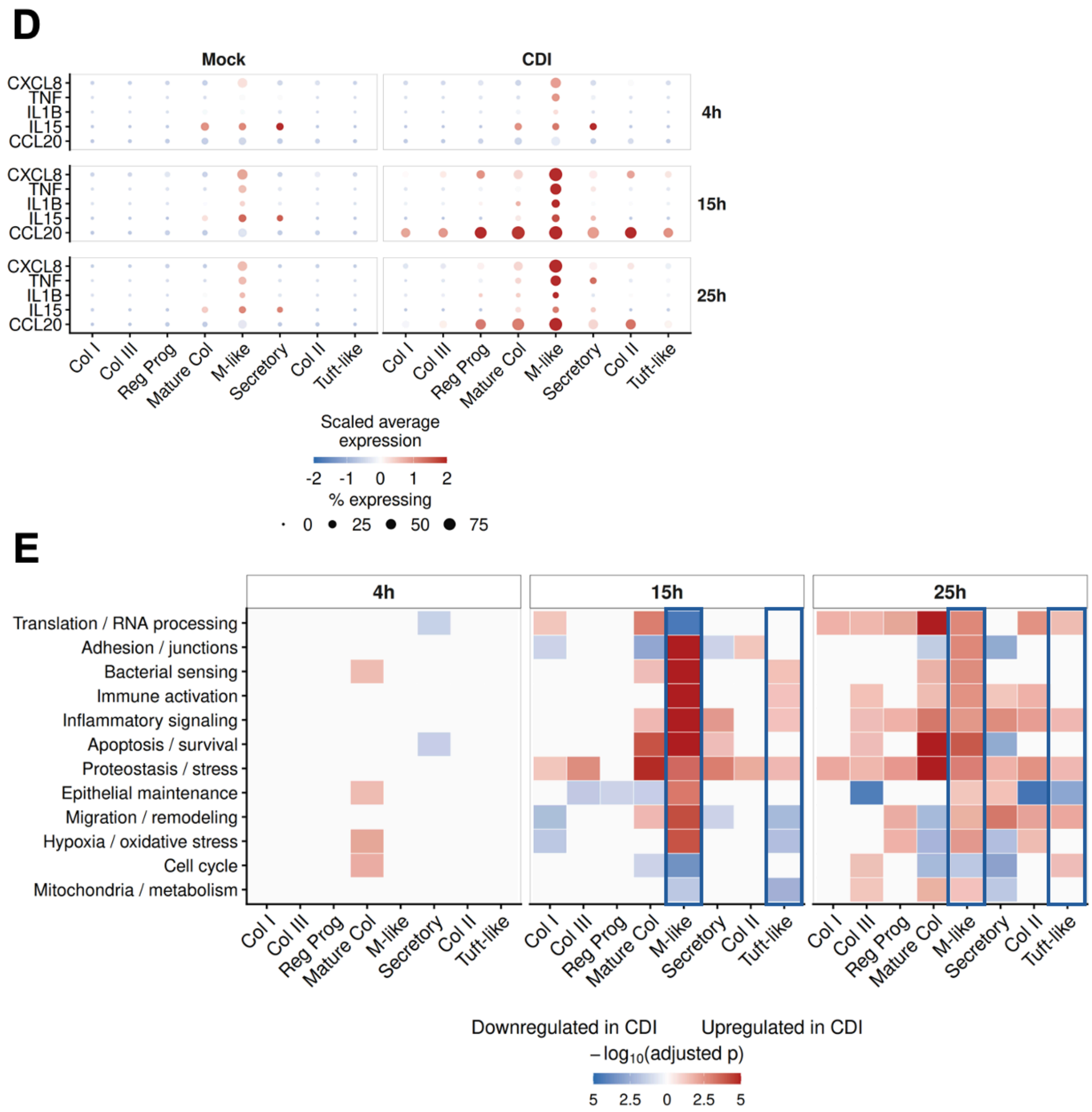

Figure S5. Responses by cell state to CDI in transcript complexity, mitochondrial burden, and injury-response programs, related to Figure 5

(A) Distribution of Decascript, Hectoscript, and Kiloscript cells across cell states, timepoints, and samples.

(B) Percentage of high-mito cells, defined as cells with mitochondrial read ratio >40%, across cell states, timepoints, and samples.

(C) Distribution of Decascript, Hectoscript, and Kiloscript classes among high-mito cells across cell states, timepoints, and samples.

(D) Dot plot of *CXCL8*, *TNF*, *IL1B*, *IL15*, and *CCL20* expression across cell states, timepoints, and samples. Dot size indicates the percentage of cells expressing each gene and color indicates scaled average expression.

(E) Heatmap of representative GO categories after excluding GO associated with *CCL20*. For each category, cell state, and timepoint, the most statistically significant GO term is shown. Values represent −log10(adjusted p), with red indicating enrichment among genes upregulated in CDI and blue indicating enrichment among genes downregulated in CDI.
